# Fundamental Issues of Melatonin-Mediated Stress Signaling in Plants

**DOI:** 10.3389/fpls.2016.01124

**Published:** 2016-07-27

**Authors:** Haitao Shi, Keli Chen, Yunxie Wei, Chaozu He

**Affiliations:** Hainan Key Laboratory for Sustainable Utilization of Tropical Bioresources, College of Agriculture, Hainan University, HaikouChina

**Keywords:** melatonin, stress signaling, abiotic stress, biotic stress, mechanism

## Abstract

As a widely known hormone in animals, melatonin (*N*-acetyl-5-methoxytryptamine) has been more and more popular research topic in various aspects of plants. To summarize the these recent advances, this review focuses on the regulatory effects of melatonin in plant response to multiple abiotic stresses including salt, drought, cold, heat and oxidative stresses and biotic stress such as pathogen infection. We highlight the changes of endogenous melatonin levels under stress conditions, and the extensive metabolome, transcriptome, and proteome reprogramming by exogenous melatonin application. Moreover, melatonin-mediated stress signaling and underlying mechanism in plants are extensively discussed. Much more is needed to further study in detail the mechanisms of melatonin-mediated stress signaling in plants.

## Introduction

*N*-acetyl-5-methoxytryptamine (melatonin) was first identified in the pineal gland of cow (Lerner et al., 1958, 1959). Later on melatonin was also discovered in plants (Dubbels et al., 1995; Hattori et al., 1995). Thereafter, melatonin has been identified in almost all plant species, although with different concentrations, including model plants (*Arabidopsis*, rice, tobacco), fruits (banana, cucumber, apple, beestrawberry), and so on (Arnao and Hernández-Ruiz, 2014, 2015; Reiter et al., 2001, 2014, 2015; Van Tassel et al., 2001; Simopoulos et al., 2005; Tan et al., 2007, 2012, 2014; Shi and Chan, 2014; Shi et al., 2015b,d,e,a,c,f). In the meantime, melatonin biosynthetic and metabolic pathways in plants have been revealed (Kang et al., 2010; Tan et al., 2012, 2014; Arnao and Hernández-Ruiz, 2014, 2015; Wang L. et al., 2014; Wang P. et al., 2014; Zuo et al., 2014; Reiter et al., 2015; Hardeland, 2016). Melatonin biosynthesis begins from tryptophan through four sequential enzyme reactions, involving tryptophan decarboxylase (TDC), arylalkylamine *N*-acetyltransferase (AANAT)/serotonin *N*-acetyltransferase (SNAT), tryptamine 5-hydroxylase (T5H), *N*-aceylserotonin methyltransferase (ASMT)/hydroxyindole-*O*-methyltransferase (HIOMT) (Tan et al., 2016; Wei et al., 2016). Thereafter, melatonin is converted to 2-hydroxymelatonin by melatonin 2-hydroxylase (M2H) (Byeon et al., 2015).

Based on previous studies using exogenous melatonin treatment or transgenic plants with higher or lower melatonin levels, some more general comprehension has been achieved as to the involvement of the compound in seed germination, root development, fruit ripening, senescence, yield, circadian rhythm, stress responses (Kolář and Macháčkova, 2005; Posmyk et al., 2008, 2009a,b; Li et al., 2012, 2015; Wang et al., 2012, 2013, 2015; Park et al., 2013; Yin et al., 2013; Zhang et al., 2013; Zhao et al., 2013; Bajwa et al., 2014; Lee et al., 2014, 2015; Zhang H. J. et al., 2014; Zhang N. et al., 2014; Liang et al., 2015; Byeon and Back, 2016). Considering the new advances in recent 5 years (Tan et al., 2012, 2014, 2015; Lee et al., 2014, 2015; Kaur et al., 2015; Reiter et al., 2015), we focus on the regulatory effects of melatonin in plant responses to multiple abiotic stress factors and plant–pathogen interactions (**Table 1**).

**Table 1:** The functions of melatonin in plant stress responses.

Plant species	Stress responses	Melatonin treatment or transgenic plants	References
*Arabidopsis*	Cold stress	Melatonin treatment	Bajwa et al., 2014; Shi and Chan, 2014
*Arabidopsis*	Disease resistance against *Pseudomonas syringe* pv. tomato	Melatonin treatment and transgenic plants	Lee et al., 2014, 2015; Lee and Back, 2016; Qian et al., 2015; Shi et al., 2015a,c, 2016; Zhao et al., 2015a
*Arabidopsis*	Leaf senescence	Melatonin treatment	Shi et al., 2015d
*Arabidopsis*	Thermotolerance	Melatonin treatment	Shi et al., 2015e
*Arabidopsis*	Salt and drought stresses	Melatonin treatment	Shi et al., 2015c
*Arabidopsis*	Oxidative stress	Melatonin treatment	Weeda et al., 2014; Wang et al., 2015
Bermudagrass	Salt, drought and cold stresses	Melatonin treatment	Shi et al., 2015b
Bermudagrass	Oxidative stress	Melatonin treatment	Shi et al., 2015f
*Nicotiana benthamiana*	Disease resistance against *Pseudomonas syringe* pv. Tomato	Melatonin treatment	Lee et al., 2014
*Lupinus albus*	Disease resistance to fungal infection (*Penicillium* spp.)	Melatonin treatment	Arnao and Hernández-Ruiz, 2015
Rice	Salt and cold stresses	Transgenic plants	Kang et al., 2010; Byeon and Back, 2016
Rice	Herbicide-induced oxidative stress	Transgenic plants	Park et al., 2013
Rice	Cadmium stress	Transgenic plants	Byeon et al., 2015
Rice	Leaf senescence and salt stress	Melatonin treatment	Liang et al., 2015
*Malus*	Disease resistance to Marssonina apple blotch	Melatonin treatment	Yin et al., 2013
*Malus*	Salt stress	Melatonin treatment	Li et al., 2012
*Malus*	Drought stress	Melatonin treatment	Li et al., 2015
*Malus*	Senescence	Melatonin treatment	Wang et al., 2012, 2013; Wang P. et al., 2014
Cucumber	Chilling stress	Melatonin treatment	Posmyk et al., 2009a
Cucumber	Salt stress	Melatonin treatment	Zhang H. J. et al., 2014
Cucumber	Drought stress	Melatonin treatment	Zhang N. et al., 2014
Red cabbage	Copper ion	Melatonin treatment	Posmyk et al., 2008, 2009b
Tomato	Drought stress	Transgenic plants	Wang L. et al., 2014

## Melatonin-Mediated Stress Responses

Secondary messengers including calcium and hydrogen peroxide (H_2_O_2_) play essential roles in plant stress responses by linking upstream receptors and activating downstream signal transduction (Shi et al., 2015b,d,e,a,c,f; Zhang et al., 2015). It has been shown that nearly all stresses including salt, drought, cold, heat, zinc sulfate, H_2_O_2_, anaerobic, pH, pathogen, and senescence can cause a rapid and massive up-regulation of melatonin production in various plants (Tan et al., 2012, 2014; Reiter et al., 2015; Shi et al., 2015b,d,e,a,c,f), indicating the possible role of melatonin as an important messenger in plant stress responses.

Most of previous studies focused on the effect of melatonin on reactive oxygen species (ROS) metabolism, as well as the alleviation of stress-induced ROS production and the activation of antioxidants in melatonin-conferred stress resistance in plants (Zhang et al., 2015). In recent years, more and more studies have extended our understanding on the molecular mechanisms of melatonin-mediated stress responses in plants. Based on previous studies, plant transcription factors play important roles in plant stress responses, by directly regulating the transcription of stress-responsive genes and through acting in cross-talk between multiple signaling pathways (Reiter et al., 2014, 2015; Shi et al., 2015b,d,e,a,c,f). In *Arabidopsis*, we have found that four transcription factors including *Arabidopsis thaliana* Zinc Finger protein 6 (ZAT6) (Shi and Chan, 2014), Auxin Resistant 3 (AXR3)/Indole-3-Acetic Acid inducible 17 (IAA17) (Shi et al., 2015d), class A1 Heat Shock Factors (HSFA1s) (Shi et al., 2015e), and C-repeat-Binding Factors (CBFs)/Drought Response Element Binding 1 factors (DREB1s) (Shi et al., 2015c), are involved in melatonin-mediated signaling. Briefly, AtZAT6-activated CBF pathway is essential for melatonin-mediated freezing stress response (Shi and Chan, 2014); AtIAA17-activated senescence-related *Senescence 4* (*SEN4*) and *Senescence-Associated Gene 12* (*SAG12*) transcripts may contribute to the process of natural leaf senescence (Shi et al., 2015d); HSFA1s-activated transcripts of *HSFA2*, *Heat-Stress-Associated 32* (*HSA32*), *Heat Shock Protein 90* (*HSP90*), and *HSP101* may contribute to melatonin-mediated thermotolerance (Shi et al., 2015e); AtCBFs-mediated signaling pathway and sugar accumulation may partially be involved in melatonin-mediated stress response (Shi et al., 2015c). Moreover, the diurnal changes of *AtCBF/DREB1s* expression may be regulated by the corresponding change of endogenous melatonin level and be involved in diurnal cycle of plant immunity (Shi et al., 2016). Thus, these transcription factors may play important roles in melatonin-mediated stress responses in plants.

Salicylic acid (SA) and NO are required small molecules for plant disease resistance, and SA-deficient plants (*NahG* overexpressing plants) and NO deficient mutants (*noa1* and *nia1nia2*) show increased sensitivity to bacterial pathogen. Moreover, both of SA and NO confer enhanced disease resistance against bacterial pathogen in *Arabidopsis*, and the cooperation between them plays important roles in plant innate immunity (Shi et al., 2012). Recently, we also found that melatonin treatment increases the accumulation of sugars and glycerol, and the elevated sugars and glycerol thereafter increase the endogenous NO level, which confers an enhanced innate immunity against bacterial pathogens via a SA and NO-dependent pathway in *Arabidopsis* (Qian et al., 2015; Shi et al., 2015a,f). Consistently, Yin et al. (2013) showed that melatonin improves Malus resistance to *Marssonina apple blotch*, and Lee et al. (2014, 2015) found that melatonin confers disease resistance against pathogen attack in *Arabidopsis* and tobacco, which may be related with endogenous SA level. Zhao et al. (2015a) found that exogenous melatonin regulates carbohydrate metabolism, increases cell wall invertase (CWI), increases production of sucrose, glucose, fructose, cellulose, xylose and galactose, and cellose deposition during pathogen infection. They also found that melatonin-mediated sugar metabolism, especially its metabolites exert significant promotional and inhibitory effects, for instance on the growth of maize seedling, as was demonstrated by treatment with different doses of exogenous melatonin (Zhao et al., 2015b). Together with previous studies suggesting that sugars are functional, well compatible solutes for osmotic adaptation in response to abiotic stress, being also involved in the protection against bacterial pathogens (Thibaud et al., 2004; Shi et al., 2015a,f; Tsutsui et al., 2015), the above studies highlight the important roles of sugar metabolism in complex plant stress responses. Recently, Lee and Back (2016) found that the mitogen-activated protein kinase (MAPK) signaling through MAPK kinase (MKK) 4/5/7/9-MPK3/6 cascades are also required for melatonin-mediated innate immunity in plants.

**FIGURE 1:**
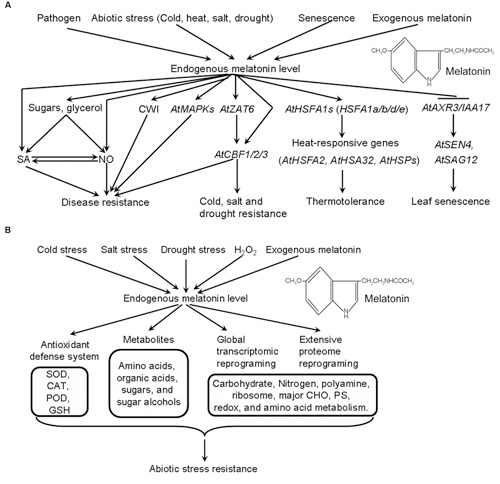
**Hypothetical model explaining melatonin-mediated stress responses in *Arabidopsis***(A)** and bermudagrass (B). (A)** Under various stress conditions, endogenous melatonin levels are quickly and significantly increased. Thereafter the induction of melatonin increases the transcripts of some stress-related transcription factors (*AtZAT6*, *AtCBFs*, *AtHSFA1s*, and *AtAXR3/IAA17*), activates MAPK signaling, CWI, and vacuolar invertase (VI), up-regulates carbohydrate metabolism especially the sugars. These affections in turn result in improved stress resistance in *Arabidopsis*. **(B)** In response to abiotic stress, endogenous melatonin levels are significantly induced. The induction of melatonin increases the activities of antioxidant defense system, triggers the extensive reprogramming of primary metabolites, transcriptome and proteome, resulting protective stress responses in bermudagrass. CAT, catalase; SOD, superoxide dismutase; POD, peroxidase; GSH, glutathione; CHO, carbohydrate; PS, photosynthesis.

Based on these results, a hypothetical model explaining melatonin-mediated signaling in *Arabidopsis* is proposed (**Figure 1A**). Under various stress conditions, endogenous melatonin levels are quickly and significantly increased. As a consequence, the induction of melatonin increases the transcripts of some stress-related transcription factors (*AtZAT6*, *AtCBFs*, *AtHSFA1s*, and *AtAXR3/IAA17*) and the underlying down-stream genes, activates MAPK signaling, CWI and vacuolar invertase (VI), up-regulates carbohydrate metabolism especially the sugars. These induced affects in turn result in improved stress resistance in *Arabidopsis*.

With the development of omics, several studies indicated that melatonin triggers extensive reprogramming of primary metabolites, transcriptome, and proteome in plants, further confirming its involvement in plant signal transduction. Weeda et al. (2014), Liang et al. (2015), and Shi et al. (2015b) identified 1308 differentially expressed genes (DEGs) (566 up-regulated genes and 742 down-regulated genes), 3933 DEGs (2361 up-regulated genes and 1572 down-regulated genes) and 457 DEGs (191 up-regulated genes and 266 down-regulated genes) by exogenous melatonin treatment in *Arabidopsis*, bermudagrass and rice, respectively. Wang P. et al. (2014) and Shi et al. (2015f) identified 309 and 63 differentially expressed proteins (DEPs) after exogenous melatonin treatment in apple and bermudagrass, respectively. MapMan and gene ontology (GO) analyses found that that several pathways were enhanced by melatonin treatment in bermudagrass, including nitrogen-metabolism, polyamine metabolism, major carbohydrate (CHO) metabolism, hormone metabolism, metal handling, photosynthesis (PS), redox status, and amino acid metabolism. Notablly, all these transcriptome and proteome studies identified a large number of transcription factors as DEGs or DEPs, the functional identification of these DEGs or DEPs may provide more valuable clues into melatonin-mediated signaling. Additionally, both Wang P. et al. (2014) and Shi et al. (2015f) indicated the possible role of melatonin in epigenetic modification in plants. Based on our previous studies (Shi et al., 2015b,f), we also propose a hypothetical model explaining melatonin-mediated stress responses in bermudagrass (**Figure 1B**). In response to abiotic stress, endogenous melatonin levels are significantly induced. The induction of melatonin activates antioxidant defense system, triggers the extensive reprogramming of primary metabolites, transcriptome, and proteome, resulting protective stress responses in bermudagrass. The “omics” approaches can give some clues about the effect of melatonin on plants, focusing on the extensive reprogramming of gene transcripts, protein expression and metabolites, as well as the relationship among them. This is just the beginning to reveal melatonin signaling in plants, many questions need to be investigated, including the crosstalk between melatonin and other phytohomones, the interaction between melatonin and primary or secondary metabolism.

## Conclusion and Perspectives

The objective of this review is to update the research on melatonin-mediated stress signaling, and to encourage plant researches to dissect further molecular mechanism and signaling pathway. Although melatonin has continuously drawn the attentions of plant biologists and some advances have been made in recent years, melatonin-mediated complex signaling pathways are largely unknown. Since melatonin shares the common substrate (tryptophan) with IAA, the cross-talk between melatonin and auxin signaling pathways needs to be further investigated (Arnao and Hernández-Ruiz, 2014, 2015). Moreover, unlike for animals (Jackers et al., 2008; Yu et al., 2014), no specific melatonin-associated phenotype and no melatonin receptor have been characterized in plants. Thus, these open questions still prevent a full understanding of melatonin signaling in plants (Reiter et al., 2015; Zhang et al., 2015). Therefore, the identification of melatonin receptor or sensor and the establishment of molecular link between melatonin sensing and the regulators for plant stress responses will be an important next step.

Moreover, several fundamental issues need to be resolved. How is endogenous melatonin production regulated? How to perceive and transfer melatonin signaling in plant cells? What are the major or limiting steps in melatonin signaling transduction in plants? Which genes are specifically regulated by melatonin and underlying signaling pathways? Together with the development of more new techniques, further studies will shed more light on the global involvement of melatonin in plants and underlying signaling pathway.

## Author Contributions

HS initiated this project, wrote and revised the manuscript, KC, and YW wrote the manuscript, CH provided suggestions and revised the manuscript. All authors approved the manuscript and the version to be published, and agreed to be accountable for all aspects of the work in ensuring that questions related to the accuracy or integrity of any part of the work are appropriately investigated and resolved.

## Conflict of Interest Statement

The authors declare that the research was conducted in the absence of any commercial or financial relationships that could be construed as a potential conflict of interest.
